# The Role of Vitamin D in Diabetes and Cardiovascular Disease: An Updated Review of the Literature

**DOI:** 10.1155/2015/580474

**Published:** 2015-10-20

**Authors:** Dimitrios Papandreou, Zujaja-Tul-Noor Hamid

**Affiliations:** Department of Natural Sciences and Public Health, Zayed University, Abu Dhabi, UAE

## Abstract

The dietary reference values for Vitamin D were set primarily considering its role in bone health, but with the discovery of Vitamin D receptors throughout body tissues, new links with other health conditions are now studied, such as for diabetes and cardiovascular diseases (CVD). This paper shall analyze and examine all new research studies carried out, especially in 2013–2015 regarding diabetes mellitus (DM) and cardiovascular diseases (CVD). Vast research has been carried out to establish strong relationship between Vitamin D serum levels, supplementation, diabetes, and CVD. However, the results from researches identified in this paper are disputable. Benefits of Vitamin D adequate levels were recognized from gestational period until later in disease development such as diabetes and/or CVD, but since not all studies are in agreement further investigation is suggested. Researches conducting large randomized controlled trials, exploring range of supplement doses, with variable baseline serum Vitamin D levels, and inclusion of array of associated parameters, are still required to conduct large-scale analysis and draw conclusion as a risk factor. Until then it is possible to conclude that maintenance of serum Vitamin D levels holds advantageous aspects in diabetic and cardiovascular conditions, and people should strive to attain them.

## 1. Introduction

Vitamin D, also called calciferol, exists in two major forms: Vitamin D2 (ergocalciferol, which is largely ingested) and Vitamin D3 (cholecalciferol, which is synthesized in human body). Both forms are inactive form, which are converted into active form, exhibiting identical responses, by two enzymatic hydroxylation reactions, first in liver forming 25-hydroxyvitamin D mediated by 25-hydroxylase and second in kidney mediated by 1 *α*-hydroxylase forming the final activated product calcitriol (1,25 dihydroxyvitamin D). Sunshine Vitamin formerly attributed with bone health is now under rigorous investigation due to expression of Vitamin D receptors (VDR) found commonly in body tissues regulating gene transcription of many inflammatory factors and immune cells expression that could potentially contribute to chronic disease prognosis, recovery, or mortality [1].

Vitamin D RDA values were set for young and adult population to acquire certain serum levels of Vitamin D, which were supported by data on osteomalacia, and for older population the recommendations also considered the fracture risk. Based on Endocrine Society's recommendation, Vitamin D deficiency should be defined as 25(OH)D of < 20 ng/mL while Vitamin D insufficiency is now recognized as 25(OH)D of 21–29 ng/mL [2]. The recommended dietary intake (RDI) for patients with risk for Vitamin D is 400 IU/d for 0–12 months, 600 IU/d for ages 1–70 years, as well as for pregnant and lactating women, and 800 IU/d for ages 71 years and older, with measurement of 25(OH)D serum level as best indicator for Vitamin D cutaneous synthesis and total intake [1]. However, with concerns about sun exposure, committee assumed conditions of minimal exposure to sunlight to describe relationship between intake and serum 25(OH)D levels; therefore, it requires adjustment of values to populations exposure to sun. The potential contribution from body stores in their report is still uncertain [1].

Cutaneous synthesis of Vitamin D is subject to a number of limitations, as excess exposure can lead to photodegradation to avoid toxicity, or other factors, such as latitude, time of exposure, skin pigmentation, obesity, and season, can all affect the synthesis [1]. To explain the effect of latitude on T1DM, study linking them has been carried out too recently [3]. Reasons that can attenuate endogenous production of Vitamin D through skin are generally lifestyle factors, such as air pollution, confined outdoor time, completely covering body during limited exposure time, and use of sunscreen [4]. In Korean adults, these factors further included older age, male sex, eating breakfast regularly, consumption of dairy and fatty fish, and use of Vitamin D-containing supplements in a positive correlation, whereas frequent consumption of instant noodles and sugar-sweetened beverages was linked negatively [5]. Among nonmodifiable factors are skin conditions as ichthyosis [3], and variants near genes involved in cholesterol synthesis (DHCR7), hydroxylation (CYP2R1, CYP24A1), and Vitamin D transport (GC) [6].

The central function of Vitamin D is elevation of plasma calcium and phosphate levels for bone health. However, Vitamin D receptors (VDR) are also found in nucleus of many tissues, which regulate several hundred genes throughout the body or as much as 5% of human genome [1]. Biological role of Vitamin D is plausible, as evidence from recent research in the following text shall point out relationship between insulin action and Vitamin D serum levels. The fostering mechanism could be either direct, that is, when VDR expression affects local production of 1,25(OH)_2_D_3_ in pancreatic *β* cells, or indirect via regulation of calcium homeostasis and calcium flux through membranes [7]. Positive effects of Vitamin D supplementation are also registered in some cases, which has further invigorated the need to investigate not only serum Vitamin D adequacy, but also the efficacy of supplements in acquiring such levels [8]. The other major investigation regarding role of Vitamin D is in development of cardiovascular diseases. Vitamin D deficiency has been found to contribute to various cardiac conditions, such as hypertension, coronary artery disease, stroke, and atherosclerosis [9–11]. Some studies consider evidence as enough to establish low Vitamin D level as CVD risk factor [12, 13], but further trials are still needed. Thus, this paper shall also identify the latest research and analysis carried out linked to serum Vitamin D levels or supplementation in cardiovascular diseases.

## 2. Vitamin D and Diabetes

Abundance of Vitamin D receptors in body tissue other than just in bone deviated attention from bone disease to chronic conditions. Clinical trials were performed extensively at different levels of diabetes mellitus stages to observe the role of Vitamin D status and to study efficacy of Vitamin D supplementation. Maintaining Vitamin D at adequate levels can be a useful preventive technique, since Vitamin D status in healthy adults was inversely associated with future risk of type 2 diabetes [14]. Significant negative correlation between 25(OH)D and HbA1c was also observed when compared between diabetic and nondiabetic patients [15]. Vitamin D dose in initial years of life is shown to reduce risk of future development of disease modulated by immune protective effects [16].

A metastudy by Forouhi et al. [17] found a strong inverse association between baseline 25(OH)D and incidence of type 2 diabetes. Vitamin D supplementation also improves HbA1c value for pediatrics with type 1 diabetes when treated with 300,000 IU single dose intramuscular injection of Vitamin D along with 40 mg/Kg/day of calcium divided into 2 doses [8]. A significant inverse relationship between Vitamin D status and insulin resistance (IR) was also observed, independent of adiposity, in Korean adolescents [18]. A recent meta-analysis that included 23 studies found that serum 25(OH)D was significantly lower in patients with type 1 DM than in healthy controls [19, 20]. On the other hand, not all studies support the result [21], along with limitation of another review study, it was concluded from the assessment of 17 randomized control trials and 7 longitudinal studies that Vitamin D supplementation did not improve hyperglycemia, beta cell secretion, or insulin sensitivity [22].

### 2.1. Supplementation Studies on Vitamin D

The review study that included 17 randomized control trials mentioned above had many limitations as most studies used daily doses less than 2000 IU to above 5000 IU of Vitamin D2 or D3. The most important limitations were the small sample groups and heterogeneity in ethnicity and baseline levels [22]. This surely highlights the need of large randomized control trials with long-term follow-up tests. Race and gender differences were also observed as far as serum Vitamin D levels and its effect in diabetes are concerned. Low 25(OH)D concentrations were found to be associated with diabetes among White but not Black people for which variation in SNP (Single Nucleotide Polymorphism) in genotype linked with either high or low Vitamin D binding protein was held accountable [23]. In newly diagnosed type 2 diabetes female patients serum 25(OH)D is associated with insulin sensitivity and *β*-cell function, but this association was ambiguous in males [24].

Significant influence was not observed on weight, fat mass, or waist circumference in a randomized double blind clinical trial with Vitamin D3 supplementation of 6000 IU of Vitamin D3/d (3 months) followed by 3000 IU/d in experimental group, and 2200 IU/d for follow-up in 6 months for both experimental and placebo groups in a recent study on type 2 diabetic obese UAE national patients [25]. Another study reported improvement in Vitamin D status and increased insulin secretion, but not insulin resistance, blood pressure, inflammation, or HbA1c in double blind randomized trial with 16 subjects in which 8 patients received 280 *μ*g daily for 2 weeks, 140 *μ*g daily for 10 weeks of colecalciferol [26]. A study by Heshmat et al. [27] carried out with 42 diabetic patients found no relationship between Vitamin D supplement and change in diabetes status (when observed for 3 months) with administration of single intramuscular injection of 300,000 IU of Vitamin D3, but Kuchay et al. [28] showed positive benefits of Vitamin D supplementation for 1 year on fasting plasma glucose, 2-h plasma glucose, and A1C levels with 137 subjects randomized to receive 60,000 IU for 4 weeks followed by 60,000 IU monthly of cholecalciferol. Type of Vitamin D supplement in effectiveness of supplementation was also noticed in few studies [29]. A meta-analysis of randomized control trials indicated that Vitamin D3 is more efficacious in raising serum 25(OH)D concentrations than Vitamin D2 [29]. At this point, it can be considered that more frequent dosage is effective in achieving benefits from supplement, as studies with single dose injection and low dose did not report efficacy of drug. Further studies investigating this opinion would be of great potential in the future.

It has been reported that aggressive treatment of Vitamin D supplementation allows improvement in serum Vitamin D levels in some studies, but it does not raise 25(OH)D status to sufficient or target levels [25, 30]. 53 patients were given ergocalciferol (D2) 50,000 IU daily over 10 days (500,000 IU) followed by Calcichew D3 (calcium carbonate/cholecalciferol) BID (~24,000 IU cholecalciferol/month); 94 patients were given cholecalciferol (D3) 40,000 IU daily over 10 days (400,000 IU) followed by calcium rich D3 BID (~24,000 IU cholecalciferol/month), or cholecalciferol 40,000 IU daily over 10 days (400,000 IU) followed by cholecalciferol 40,000 IU monthly to 97 patients [30]. Rise in 25(OH)D status to “sufficient” levels was concluded to be inadequate in large proportions of individuals in this study [30]. Vitamin D supplementation requires longer duration trials, with variable doses, and array of characteristic variables to establish stronger stance. Trials comparing the efficacy of Vitamin D2 and that of Vitamin D3 supplement in most similar conditions studying dosage periods as well would be quite valuable.

Generally, Vitamin D levels are safe and with many health benefits associated with gestational and type 1 diabetes mellitus. Vitamin D supplementation during pregnancy is shown to have positive birth weight and length outcomes; however, there is significant need for randomized controlled trials to establish efficacy and safety of Vitamin D supplementation as concluded from meta-analysis [31, 32]. Vitamin D supplementation in early pregnancy was also shown to have inverse association with GDM (gestational diabetes mellitus) risk [33]. Supplementation in earlier life is further linked with reduced risk of type 1 diabetes, but studies to suggest association between maternal intake and type 1 diabetes in offspring are inadequate [34].

### 2.2. Genetic Factors of Vitamin D Related to Diabetes

Genetic factors that may affect the Vitamin D levels in body were also explored for their link with diabetes. DHCR7 encoding 7-dehydrocholesterol reductase enzyme, which converts 7-dehydrocholesterol to cholesterol, a precursor of Vitamin D3, playing a role in endogenous production of Vitamin D was studied. Genetic variants of DHCR7 were significantly associated with increased risk of type 2 diabetes, whereas CYP2R1 was not in a Danish study with 96,423 participants genotyped [35]. Allele frequencies of 18 SNPs derived from CYP2R1, GC, and DHCR7/NADSYN1 investigated in South Asians, South-East Asians, and Arabs living in Kuwait indicated significant association between the GC (rs2282679 and rs7041), CYP2R1 (rs10741657), SNPs, and 25(OH)D levels [36]. Another European cohort (IMPROVE) based study inclusive of 3,418 individuals, of whom 929 had type 2 diabetes Single Nucleotide Polymorphisms (GC; rs2282679 and rs7041) and 7-dehydrocholesterol reductase/NAD synthetase-1 (DHCR7; rs12785878 and rs3829251), was investigated and found to have negative association with 25(OH)D levels, with differences in association significances between type 2 diabetic and nondiabetic individuals. Furthermore, rs3829251 (DHCR7) influenced progression of subclinical atherosclerosis measured in a relationship dependent on type 2 diabetes status, but independent of 25(OH)D levels [37].

Involvement of VDR in T-cells immune response to antibacterial infection, regulation of complex set of regulatory factor, and control over adverse inflammatory adaptive immunity support Vitamin D's role in decidual immunity and possibly as future immunotherapy [38–41]. Mechanism through which Vitamin D was involved was also widely studied, and specific genes and their polymorphic conditions were discovered for Vitamin D receptors genes that accounted for some degree of risk. Vitamin D downregulates NF-*κ*B and its downstream inflammatory cytokines expression and upregulates PPAR-*α*, acting as antagonist, promoting *β*-oxidation, and reducing triglycerides level. It increases the expression of CPT-1, a rate limiting enzyme in mitochondria, which allows for transport of free fatty acids into mitochondria for lipid oxidation, thus reducing lipid deposition [42]. Vitamin D level was negatively related with serum CRP, TNF-*α* and IL-6 levels and urinal inflammation factors in patients with type 1 diabetes as well [43]. Investigations exploring specific genes, involved in diabetes, commonly hold variable alleles of the same gene accountable for risk and complications in this disease. T and b, TaqI and BsmI alleles were suggested to be protective for developing T1DM in Koreans [44]. VDR FokI polymorphism is linked to T1DM [45], or to microvascular complication [46]. Study on VDR polymorphism for the whole gene indicated susceptibility for type 1 diabetes [47]. However, meta-analysis of studies since 1998 until 2013 concludes that individual VDR polymorphisms seemed not to be associated with T1D risk, but haplotypes contributed significantly to disease susceptibility [48]. Further recognition sites were found on ApaI and TaqI for VDR gene in Egyptian type 1 DM patients [49]. VDR rs2228570 was also suggested as a good candidate for biomarker of diabetic retinopathy in Han Chinese T2DM patients due to the association found with the T allele [50]. Certain VDR genes were further investigated for 1692 children from DAISY cohort, and an association with type 1 diabetes progression and polymorphism of genes was concluded; however, large cohort studies are required to replicate such data [51]. Such link of genes is also discussed in regard to diabetic nephropathy [52], where ACR (Albumin-Creatinine Ratio) is linked with progression from prediabetes to diabetes [53]. Moreover, VDR polymorphism of functional SNP 2228570 C > T (FokI) of T allele can further influence the severity of metabolic syndrome in T2D patients amongst Egyptian population [54].

Vitamin D involvement in diabetic complications attenuation was also reported in some studies. An inverse and independent relationship between circulating 25(OH)D levels and the prevalence of microvascular complications in patients with type 2 diabetes was shown [55]. Low Vitamin D status is characteristically associated with advanced diabetic nephropathy [56]. Duration of diabetes and Vitamin D deficiency were independent determinants of diabetic retinopathy in young Japanese type 1 diabetic patients [57], but there has also been report representing no link [58]. Good status of Vitamin D could delay diabetic nephropathy [43]. Suggested mechanism is through protection of podocyte, shown in mice experiment, which determines the outcome of diabetic nephropathy, or by reducing the renal fibrosis [59, 60]. Data on supplemental use of 22-oxacalcitriol, Vitamin D receptor activator, further suggests improved bone formation due to antioxidative property of Vitamin D in diabetic bone disease [61]. Recently, Vitamin D is also shown to be a potential protective factor for cognitive impairment in DM patients [62]. Both hypovitaminosis D and diabetes affect cardiovascular factors and could further mediate development of CHD, as suggested by a study on 798 patients [63].

Vitamin D controls the macrophage adhesion and migration, otherwise induced by its deficiency, which is a critical factor in atherosclerosis progression [64]. Vitamin D might also pose as a factor in development of vascular problems. 25(OH)D levels were found to be lowest in T2DM patients with lower extremity arterial disease (LEAD) compared to T2DM without LEAD (keeping in consideration the study limitations) [65]. Studies registered positive effects of Vitamin D sufficiency or supplementation usually, commonly as a consequence of improved diabetic parameters. To draw strong conclusion from such data, replication is crucial, since baseline characteristic was usually dissimilar, or trials dealing with same conditions were inadequate. Even to perform meta-analytic studies, extensive research is needed to determine reliable results from latest technology. The interaction of Vitamin D receptors with body's immune and inflammatory response explained through experimentation and registered through biomedical analysis surely suggests an important role, especially when the parameters identified are associated with the most common chronic conditions. Vitamin D supplementation in initial years of life or during gestational diabetes have showed positive results, but due to inadequate controlled trials carried out, further experimentation considering range of BMI, age, Vitamin D dose, duration, and so forth is imperative to draw clear line for dose efficacy and necessity. In adults much stronger influences need to be controlled, especially when Vitamin D plays a role in immunity, and adults have more developed immune system. Vitamin D supplementation usually improved the serum levels, but to attain target levels proved to be difficult; furthermore, since BMI could also play significant role, to establish specific dose in different conditions and over different anthropometric variables requires many trials tending to large and statistically significant data. However, the general approach that can be concluded with current results is that Vitamin D status in sufficient range holds no harm. Vitamin D consumption within normal range is considered beneficial. A summary of studies is presented in Table 1.

## 3. Vitamin D and CVD

Involvement of Vitamin D in numerous imperative metabolic pathways has been found to mediate health benefits by reducing risk through its effect on confounders in cardiovascular disease (CVD). The current trials and evidence are not adequate to establish causative link between Vitamin D deficiency and mortality from CVD [66], but it is shown in a systematic review and meta-analysis that Vitamin D3 supplementation does reduce overall mortality in older adults; however, further investigation regarding optimal dose and duration is necessary [67]. Studies on Vitamin D3 supplements, which were included in the meta-analysis, received supplementation ranging from 10 to 6000 IU/d (common administration form was tablets), and Vitamin D2 research received 208 to 4500 IU/d with not much heterogeneity to be observed with Vitamin D3. As regards Vitamin D2 supplementation, increased risks of mortality were found with lower intervention doses and shorter average intervention periods [67]. Vitamin D deficiency and mortality from CVD could not be linked as causal, possibly because Vitamin D deficiency could be a consequence of CVD leading to low plasma 25(OH)D other than being the cause of CVD [68]. This can be summarized by “resilience factor hypothesis,” which suggests that Vitamin D deficiency could be an indirect risk factor, causing fatal outcomes of the disease linked by immunosuppressive effects and inflammatory markers, rather than being direct cause of fatality [66]. In a study including 7746 US adults, Vitamin D deficiency was associated with mortality from chronic obstructive pulmonary disease (COPD) [69]. The association between Vitamin D status and COPD observed could be because COPD patients develop a low Vitamin D status as a consequence of the disease rather than Vitamin D status affecting the development and progression of the disease (as persons with COPD are more prone to Vitamin D deficiency for several reasons: the physical impairment leads to reduced outdoor activity, old age which is associated with low Vitamin status, etc.) [70]. Hence, the crucial dilemma is whether Vitamin D fosters immune response in fatal course, or if immune system fosters Vitamin D deficiency during fatal course.

Biochemical processes are further explored to assess the pathway to expand on the dilemma faced regarding the fatal course of disease. The suggested mechanism through which Vitamin D contributes to cardiovascular health is by suppressing genes involved in producing renin, thus downregulating the RAA system [71]. Hypovitaminosis of Vitamin D induces calcium deposition in vessels and soft tissues, which may also activate RAA system [71]. Vitamin D receptors involvement in inflammation further facilitates reduction of atherogenesis by downregulating proinflammatory factors and upregulating anti-inflammatory factors, thus preserving the endothelial function of vascular muscle cells [71, 72]. Other confounders of this chronic condition are also under influence of Vitamin D, such as metabolic syndrome, hypertension, hyperglycemia, hyperlipidemia, insulin resistance, and obesity for which Vitamin D is shown to be beneficial, as reviewed in [73, 74].

Results support causal effect of higher Vitamin D status on a more favorable lipid profile [75] and decreased incidence of metabolic syndrome [76], but more studies are required to establish proper causative link. Thus, mechanisms are suggested and supporting values are provided; however, science is always about experimenting and needs continuous testing to explain further. Beneficial HDL cholesterol, triglycerides, waist circumference, serum glucose, and low prevalence of metabolic syndrome were observed with higher Vitamin D concentration [77, 78].

Vitamin D supplementation might improve mortality in chronic kidney disease patients, since a study reports insulin resistance and Vitamin D deficiency as independent predictors of left ventricular hypertrophy and atherosclerosis [79]. Low 25(OH)D levels were also found to be associated with reduced incidence of CVD, much significantly for fatal cases [80]. A meta-analysis of the most well controlled randomized human trial data available however does not show benefit of Vitamin D on CVD, although small studies indicate benefits [81].

Vitamin D status was also found to have no role in the etiology of Atrial Fibrillation [82]. But augmentation index, to measure arterial stiffness, was reduced [83] in patients when supplemented (119 deficient patients randomized to receive either 50,000 international units (IU) or 100,000 IU single intramuscular Vitamin D3) and so did other mediators of arterial stiffness (62 participants received cholecalciferol 2,000 IU/day + calcium 200 mg/day, or the placebo with calcium 200 mg/day) [84]. In diabetic patients it did not show any benefits [85].

Evidence for association between Vitamin D status and ischemic stroke still remains equivocal. No association between Vitamin D status and incidence of stroke was found in some studies [86, 87], but low 25(OH)D levels as risk factor for stroke, specifically for those predisposed to high D binding protein, who thus have low bioavailability of Vitamin D, were also presented as hypothesis in one experiment with supporting outcome [10]. In Indian subjects Vitamin D deficiency was also associated with acute myocardial infarction (MI) [88]. Racial disparity was observed in heart failure cases for Vitamin D deficient patients [89]. In some conditions, such as MI, stroke, and arterial stiffness, data from recently performed experiments is not adequate to conclude, and it shall be valuable to continue recording with latest technology.

Data on blood pressure is still indecisive. In Mendelian analysis it was concluded that increased Vitamin D levels could reduce hypertension risk, but further replication of study is necessary [90]. Vitamin D supplementation for hypertensive, low Vitamin serum levels subjects did not improve their blood pressure when given either 2800 IU of Vitamin D3 per day as oily drops (*n* = 100) or placebo (*n* = 100) for 8 weeks [91]; further controlled trial is on the way to assess the link [92]. In a review conducted on Vitamin D supplementation trials, the result was inconclusive, since randomized trials with higher Vitamin D dose are potentially required to realize specific outcome [93]. Through Mendelian randomization analysis, a causal effect of Vitamin D deficiency on increased blood pressure was suggested [94]. Studies need to be replicated in an independent, similarly powered study, as some inconsistencies were present that could be attributed to the baseline differences in 25(OH)D concentrations or blood pressure, or other sources of heterogeneity between the studies.

### 3.1. Supplementation Studies on Vitamin D

Vitamin D supplementation effects were investigated too, commonly to improve serum levels and to study the effect it had for target identified in research studies. Vitamin D supplementation in deficient people, free of cardiovascular risk, improved myocardial deformation parameters and epicardial fat thickness; moreover, link between Vitamin D levels and impaired left ventricular global longitudinal strain was suggested [95]. Vitamin D replacements in type 2 diabetic patients potentially have beneficial effects on cardiovascular disease risk factors too, as indicated by research on 119 type 2 diabetic patients given calcitriol 0.5 micrograms per day for 8 weeks [96]. Vitamin D deficiency is also shown through one study, to play a role in hypertension [97]. Vitamin D supplementation could aid control of systolic blood pressure, diastolic blood pressure, and mean arterial blood pressure, as concluded from study with 42 outpatients with elevated blood pressure given one capsule containing 50 000 IU of cholecalciferol weekly in intervention group [98]. Endothelial function might be impaired in healthy but Vitamin D deficient young women, which is shown to improve with 6-month replacement therapy, possibly due to immune-modulatory effects [99]. Vitamin D might also influence efficacy of drugs, as finding from study on children with lupus assessing effect of atorvastatin on carotid IMT progression reflects that serum 25(OH)D level ≥ 20 ng/mL had less carotid IMT progression from treatment [100].

### 3.2. Vitamin D's Relation to Cardiovascular Inflammatory Conditions

CVD development and progression commonly involve atherosclerosis buildup and narrowed vessels; the following trials shall indicate the link between these conditions and Vitamin D serum status. Vitamin D deficiency may lead to coronary atherosclerosis, and plaque buildup, but study does not define relationship with the composition [101]. Meta-analysis conducted for research published on “PubMed” from 2009 onwards does not conclude beneficial effect of Vitamin D on vascular reactivity [102]. To study presence of calcific atherosclerosis or obstructive coronary artery stenosis with low Vitamin D, study inclusive of 1131 individuals was conducted; however, no association was found [103]. In 375 patients undergoing coronary angiography, Vitamin D was the most significant predictor found for coronary artery disease measured by >50% stenosis in each major coronary artery [104]. To conclude link between Vitamin D levels and coronary atherosclerosis measured by coronary artery calcium, evidence is insufficient and needs further large sample studies with proper Vitamin D assessment levels and confounders adjustment (reviewed in [105]). Carotid intima media thickness (CIMT) and arterial stiffness were not associated with Vitamin D deficiency in patients with type 2 diabetes [106], with systemic lupus erythematosus [107, 108], nondiabetic males with HIV [109], amongst juvenile and adult [110], with primary hyperparathyroidism [111], or random patients admitted to internal medicine [112]. On the other hand, serum Vitamin D level was significantly, independently associated with carotid atherosclerosis in type 2 diabetes patients, since CIMT levels and proportion of carotid plaques were higher in lowest quartile of 25(OH)D than in the highest quartile [113], or in psoriasis patients with increased risk of carotid atherosclerosis, especially those with a longer history of psoriasis [114], while the first published study on healthy postmenopausal Chinese women investigating CIMT and Vitamin D also found a similar inverse relationship [115]. Low 25(OH)D level in childhood was shown to have increased risk for carotid IMT in adulthood [116]. Study investigating relationship between higher Vitamin D levels and cardiovascular risk also reports from its 567 subjects that serum levels of 50 nmol/L and above show slight increase in IMT with increasing 25(OH)D levels [117]. Hypovitaminosis D might further be involved in thoracic atherosclerosis pathogenesis too, as lower 25(OH)D was independently associated with higher aortic IMT [118]. Studies on thromboembolism do not provide conclusive result and lack large group trials; results of studies focusing on P-selectin or hs-CRP are nonsupportive [119, 120], even when considering it as risk factor to disease [121]. However, significant inverse relationship between serum 25(OH)D level and hs-CRP levels (inflammatory marker) was also detected, and it is this inflammatory process throughout the disease which plays a role in its progression [98]. Insulin-like growth factor-1 (IGF-1), which evidentially counteracts vascular aging, circulates in blood when Vitamin D levels are low, according to Baltimore Longitudinal Study of Aging (BLSA) including 472 participants [122].

### 3.3. Genetic Factors of Vitamin D Related to Cardiovascular Diseases

Genetic factors that contribute to Vitamin D status of body were also investigated for their role in cardiovascular diseases. Single Nucleotide Polymorphisms (SNPs) in GC and DHCR7 were associated with serum levels of 25(OH)D levels, and only rs3829251 (DHCR7) influenced progression of subclinical atherosclerosis [37]. In another study inclusive of 535 individuals SNPs of CYP1A1 and CYP1B1 were identified and indicated synergistic effect on blood pressure [123]. Sixty-three male patients recruited with coronary artery disease (CAD) were genotyped for both the rs12785878 (NADSYN1) polymorphism and the rs1790349 (DHCR7). Data backs the investigated SNPs ability to predict circulating 25(OH)D levels but opposes their use as genetic markers for CAD [124]. Future studies addressing the interaction could be insightful for prevention and treatment. Many trials recognize low serum levels of Vitamin D to impact cardiovascular health negatively, whereas some still do not support, and so continuous experimentation expanding on vast range of confounders is suggestive to draw conclusion. A summary of studies is presented in Table 2.

## 4. Conclusion

This paper generally looked at researches or reviews conducted mostly during 2013 and mid-2015. Majority of results put forth suggest beneficial associations between Vitamin D, diabetic conditions, and cardiovascular diseases. And, the mechanisms defined propose maintenance of Vitamin D levels in desired range to prevent, delay, or control diabetes. Serum Vitamin D levels also improved cardiovascular function. All benefits of Vitamin D are concentrated in the role of this molecule in inflammation, immunity, and gene transcription. Use of supplement also proved to be beneficial, but to the extent of acquiring proper serum levels. To establish specific recommendation regarding their use still demands insight into the administered doses and duration, as in some studies Vitamin D supplementation doses were high, but for short time, whereas in others they were administered for few months in small doses. Thus, maintaining adequate Vitamin D levels is vital for chronic condition prevention, severity, and improvement.

Conclusive recommendation is that further trials are necessary to establish causal risk association. Many aspects of CVD, such as stroke, blood pressure, atrial stiffness, carotid intima, and media thickness, provide equivocal results. It is imperative to continue with large, long-term controlled trials, including range of disease confounders and properly identified baseline data to reach final stance. Further studies are required to establish causality, minimize population based genetic variance, harmonize baseline Vitamin D levels, and target specific groups. Prospective study considering changing Vitamin D levels year-round would eliminate certain limitations associated with study type reported in some cases.

## Figures and Tables

**Table 1:** Vitamin D and diabetes.

Study type	Subject division		Population	Vitamin D assessment/supplement	Duration	Conclusion	Citation
Cross-sectional	Non-DM T2DM patients	100 698		Vitamin D assessment & main cardiovascular risk factors	—	Prevalence of hypovitaminosis D was higher in diabetic patients with HbA1C, BMI, LDL, and triglycerides than Vitamin D counterparts. 25(OH)D may have an indirect effect mediated by cardiovascular risk factors on CHD	[63]

Cross-sectional	Controls Case, diabetic retinopathy	110 94	Chinese	Vitamin D receptor gene polymorphism investigated	—	Diabetes duration, systolic blood pressure, HbA1c, and the rs2228570 T allele were associated with increased risk of diabetic retinopathy	[50]

Prospective case-control	Control Case (deficient/insufficient serum 25(OH)D3)	30 31	China	Vitamin D supplement: case, calcitriol 0.25 *μ*g/daily Control, no dose	6 months	No effect on FBG, HbA1c, and AUCC-peptide, but reduced blood pressure, inflammation markers, and proteinuria levels after 6 months from baseline	[43]

Case-cohort	Control (non-GDM) Case (GDM)	517 135		Vitamin D assessment		Early pregnancy Vitamin D status was found to be inversely associated with GDM	[33]

Prospective cohort	White Black	8120 2102	Diverse	Vitamin D assessment	8 years	Risk association in Blacks No association in Whites	[23]

Case-control	Control Case, supplemented	68 69	India	Dose administration: 60,000 IU weekly for 4 weeks and then monthly	1 year	Reduced HbA1c levels, FPG, and 2-h plasma glucose	[28]

Randomized double blind	Control Subjects (DM patients)	21 21	Tehran	Dose administration: intramuscular injection 300000 IU of Vitamin D3	3 months	Dose of Vitamin D improved plasma level but had no effect on HbA1c	[27]

Randomized double blind	Placebo Case, T2DM Vitamin D supplemented	42 45	UAE	Vitamin D supplementation: phase 1 6000 IU Vitamin D3/day (3 months), phase 2 with 3000 IU Vitamin D3/day, and phase 3 both the arms unblinded and supplemented with 2200 IU Vitamin D3/day for 6 months	1 year	No significant influence on weight, fat mass, or waist circumference. Target levels of S-25(OH)D above 75 nmol/L in this population were not achievable	[25]

Randomized prospective	T1DM male (62%) Female (38%)	25	62% Hispanics	Vitamin D3 (20 000 IU/week) for 6 months, either immediately or after 6 months of observation	1 year	Did not affect glycaemia or markers of inflammation	[21]

Cross-sectional	Female Male	697 769	Korean	Vitamin D assessment	—	Significantly decreasing trends for fasting insulin, HOMA-IR, and IFG with increasing 25(OH)D (independent of adiposity)	[18]

Prospective case-control	Control Case (diagnosed DM)	102 102	India	Vitamin D assessment	2 years	Controls and cases were deficient in Vitamin D, but it was significantly lower in DM patients. Significant negative correlation between 25(OH)D and HbA1c was observed	[15]

**Table 2:** Vitamin D and cardiovascular diseases.

Study type	Subjects division		Population	Vitamin D assessment/supplement	Duration	Conclusion	Citation
Prospective	Female Male	28 32	Iranian	Vitamin D assessment, P-selectin, and hs-CRP levels measurement for thrombosis condition	3 months	Did not find significant relationship between Vitamin D and P-selectin and hs-CRP levels	[120]

Cross-sectional	Postmenopausal women	926	Chinese	Vitamin D assessment & carotid IMT measurement		Serum 25(OH)D inversely correlated with carotid IMT	[115]

Cross-sectional	Female (43%), male (57%)	567		Vitamin D assessment, arterial stiffness, and carotid IMT		Nonlinear relationship between 25(OH)D and IMT, and IMT increases slightly for 25(OH)D levels above 50 nmol/L	[117]

Prospective	2148		Finnish	Vitamin D assessment & carotid IMT measurement	27 years	Low Vitamin D levels were associated with increased carotid IMT in adulthood	[116]

Cross-sectional	T2DM	352	Chinese	Vitamin D assessment, carotid plaques, and carotid IMT		Serum Vitamin D independently associated with carotid atherosclerosis in T2D	[113]

Randomized controlled	T2D	415	Danish	Vitamin D assessment, carotid IMT and arterial stiffness markers, and DXA scan	4 years	25(OH)D status not associated with carotid IMT, arterial thickness, or bone health	[106]

Cross-sectional	Control Case (atherosclerosis)	110 98		Vitamin D assessment & coronary CT angiography		Relationship between low Vitamin level and coronary atherosclerosis and plaque burden, but not with morphology	[101]

Randomized controlled	Placebo Case (atorvastatin)	103 98	Diverse	Vitamin D assessment, carotid IMT progression, and secondary outcomes (cholesterol, LDL, and hs-CRP)	3 years	Baseline 25(OH)D levels ≥21 ng/mL associated with lower baseline hs-CRP levels. Vitamin D deficiency may be involved in response to atorvastatin	[100]

Randomized controlled	Placebo Case-supplement	100 100	Austria	Vitamin D supplementation for cases, 2800 IU of Vitamin D3/day for 8 weeks		Supplementation for hypertensive patients with low serum levels has no significant effect on blood pressure	[91]

Prospective	White (76%), Black (24%)	12215	Diverse	Vitamin D assessment	21 years	Association between Vitamin D and risk of heart failure found for both Black and White people, but in White people it also reflected incidence of heart failure	[89]

Cross-sectional	Controls Case (stroke patients)	70 73	Indian	Vitamin D assessment, iPTH levels, and risk factors of stroke		Vitamin D deficiency is not linked with ischemic stroke or its risk factors	[87]

Prospective	Female Male	2007 1388	Netherlands	Assessment of Vitamin D, Atrial Fibrillation (AF), and confounders	12 years	Vitamin D status was not associated with AF incidence	[82]

Cohort	9949		German	Vitamin D assessment and fatal and nonfatal CVD incidence	5 years	Relationship suggests that low 25(OH)D levels moderately increase risk of CVD, much strongly for fatal incidences	[80]

Prospective Mendelian	95766		Danish	Vitamin D assessment, genotypes for DHCR7 & CYP2R1, and mortality confounders	5–19 years	Vitamin D concentration not associated with CVD mortality but could be result of confounding factors	[68]

Cross-sectional	Without plaque With plaque	712 289	Chinese	Vitamin D assessment, carotid plaque, and carotid IMT		Serum Vitamin D levels are inversely associated with atherosclerosis. Vitamin D is also a protective factor for increased carotid IMT amongst subjects with plaque	[11]

Prospective	12158			Vitamin D assessment, stroke incidence, and rs7041, rs4588 SNPs for D binding protein were genotyped	20 years	Low 25(OH)D levels are risk factor for stroke, especially those predisposed to high DBP	[10]
